# Genetics and Epigenetics of Parkinson's Disease

**DOI:** 10.1100/2012/489830

**Published:** 2012-05-01

**Authors:** Fabio Coppedè

**Affiliations:** ^1^Faculty of Medicine, University of Pisa, 56126 Pisa, Italy; ^2^Genetics and Epigenetics of Complex Disease Program, Department of Neuroscience (DAI Neuroscience), Pisa University Hospital, Via S. Giuseppe 22, 56126 Pisa, Italy

## Abstract

In 1997 a mutation in the *a-synuclein* (*SNCA*) gene was associated with familial autosomal dominant Parkinson's disease (PD). Since then, several loci (PARK1-15) and genes have been linked to familial forms of the disease. There is now sufficient evidence that six of the so far identified genes at PARK loci (*a-synuclein, leucine-rich repeat kinase 2, parkin, PTEN-induced putative kinase 1, DJ-1*, and *ATP13A2*) cause inherited forms of typical PD or parkinsonian syndromes. Other genes at non-PARK loci (*MAPT, SCA1, SCA2, spatacsin, POLG1*) cause syndromes with parkinsonism as one of the symptoms. The majority of PD cases are however sporadic “idiopathic” forms, and the recent application of genome-wide screening revealed almost 20 genes that might contribute to disease risk. In addition, increasing evidence suggests that epigenetic mechanisms, such as DNA methylation, histone modifications, and small RNA-mediated mechanisms, could regulate the expression of PD-related genes.

## 1. Genetics of Parkinson's Disease (PD)

Parkinson's disease (PD) is a common neurodegenerative disorder affecting 1-2% of the population over the age of 65 years and reaching a prevalence of almost 4% in those aged above 85 years. Resting tremor, rigidity, bradykinesia, and postural instability are the main clinical symptoms of the disease often accompanied by nonmotor symptoms including autonomic insufficiency, cognitive impairment, and sleep disorders. The brain of PD individuals is pathologically characterized by a progressive and profound loss of neuromelanin containing dopaminergic neurons in the *substantia nigra* with the presence of eosinophilic, intracytoplasmic inclusions termed as Lewy bodies (LBs: containing aggregates of *α*-synuclein as well as other substances), and Lewy neurites in surviving neurons. Unfortunately, only some improvements of the symptoms are offered by current treatments based on levodopa and dopaminergic therapy, but there is no currently available treatment to arrest the progression of the disease [1].

 A familial history of PD is shown in approximately 20% of the cases, and in a minority of them the disease follows Mendelian inheritance patterns. Studies in PD families led to the identification of 15 PD loci (PARK1-15), and 11 genes for PARK loci have so far been described (Table 1). Although follow-up genetic studies have been inconsistent for some of them or conclusive data are still pending, there is evidence that five of those genes (*a-synuclein, parkin, PTEN-induced putative kinase 1, DJ-1, *and* leucine-rich repeat kinase 2*) cause typical PD [2], and mutations of *ATP13A2 *(PARK9) cause Kufor-Rakeb disease, an autosomal recessive parkinsonism with many other features, including pyramidal tract dysfunction, supranuclear gaze paresis, and dementia [3]. The vast majority of PD cases are however sporadic (idiopathic) forms, likely resulting from a combination of polygenic inheritance, environmental exposures, and complex gene-environment interactions superimposed on slow and sustained neuronal dysfunction due to aging [4]. 

Therefore, linkage analyses in PD families have been paralleled by several hundreds of genetic association studies in order to identify genetic variants able to modify the individual risk to develop idiopathic forms of the disease. These studies have been conducted by either the candidate gene approach or, more recently, by means of genome-wide screenings, that is, genome-wide association studies (GWASs). The genetic screening has been successful with a common high-risk locus identified (*GBA*), and many common low-risk loci (*SNCA, MAPT, LRRK2*) were recently elucidated [24]. Moreover, the application of GWAS technology and the creation of international collaborative groups sharing samples and results are continuously revealing novel variants that could contribute to disease risk [25].

The last few years have seen the growing of evidence dealing with the possible contribution of epigenetic modifications to human diseases, including among others neurodegenerative ones [26–28]. The term “epigenetics” is used to describe those mechanisms able to modify the expression levels of selected genes without necessarily altering their DNA sequence, including DNA methylation, histone tail modifications, and chromatin remodeling, as well as mechanisms mediated by small RNA molecules. Epigenetic modifications are often environmentally induced, and tissue-specific phenomena that can have similar effects to those of pathogenic mutations or functional polymorphisms, since they are able to silence, increase or reduce the expression of a selected gene in a given tissue [29]. This paper aims at describing the current knowledge on genetic alterations and epigenetic modifications likely contributing to PD pathogenesis.

## 2. Familial Parkinson's Disease

There is sufficient evidence that six of the so far identified genes at PARK loci cause inherited forms of typical PD or parkinsonian syndromes. Only two of them (*a-synuclein* and *leucine-rich repeat kinase 2*) cause autosomal dominant forms, whilst the other four (*parkin*, *PTEN-induced putative kinase 1*, *DJ-1, *and *ATP13A2*) are inherited in an autosomal recessive fashion. Additional PARK loci have been so far described (Table 1), pending identification of a causative gene or confirmation/validation of the candidate one. Moreover, in addition to the genes mapped to the sequentially numbered PARK1-15 loci, other genes, such as *MAPT*, *ATXN2*, *ATXN3*, *spatacsin*, and *POLG1 *(Table 2), are known to cause syndromes that can clinically manifest with parkinsonism as one of the symptoms [24].

### 2.1. Autosomal Dominant PD

#### 2.1.1. *α*-Synuclein (*SNCA*): PARK1 and PARK4

In 1997 Polymeropoulos et al. [5] described a mutation in the *a-synuclein *gene (*SNCA*) on 4q21 (PARK1), causing an A53T amino acidic substitution and segregating with PD in an Italian kindred and 3 unrelated families of Greek origin [5]. Two additional *SNCA *mutations (A30P and E46K) were subsequently described in other families with autosomal dominant PD [6, 7]. Moreover, a triplication of the *a-synuclein *gene (PARK4) was observed in a large family as causative of PD [8], and other PD families have been described with *a-synuclein *gene duplication and a disease course less severe of that observed in PARK4 carriers, suggesting the existence of a gene dosage effect [9]. Although *SNCA* has been the first PD gene identified, *SNCA* mutations and multiplications are both extremely rare causes of familial autosomal dominant PD [2]. *α*-Synuclein is expressed throughout the mammalian brain particularly in presynaptic nerve terminals, and mutated *α*-synuclein has an increased tendency to form aggregates critical to Lewy bodies (LBs) formation. These fibrillar aggregates are the major component of LBs in both familial and idiopathic PD, and aggregation of *α*-synuclein is thought to be a key event in dopaminergic neuronal cell death. The function of *α*-synuclein under normal physiological conditions is not yet completely clear, although there is evidence that implicates SNCA in neurotransmitter release and vesicle turnover at the presynaptic terminals [39, 40].

#### 2.1.2. Leucine-Rich Repeat Kinase 2 (*LRRK2*): PARK8

The *LRRK2 *gene was mapped on the PARK8 locus in 12q12 and was the second gene linked to autosomal dominant PD [10, 11]. Over 100 *LRRK2 *mutations have been so far described in PD families and sporadic cases, but the pathogenic role of many of them has not yet been proven (a complete list can be found at: http://www.molgen.ua.ac.be/PDmutDB/). *LRRK2 *encodes a protein named dardarin (from *dardara*, the Basque word for tremor) which contains several domains including the catalytic domain of a tyrosine kinase. The presence of a kinase domain suggests a role for dardarin in signaling cascades, likely relating to cytoskeletal dynamics [24]. The most prevalent *LRRK2 *mutation is a G2019S missense mutation occurring in 1-2% of PD patients of European origin, 20% of Ashkenazi Jewish patients, and approximately 40% of Arab Berbers with PD. The Arg1441 codon is another frequent hotspot of *LRRK2* pathogenic mutations [2].

### 2.2. Autosomal Recessive PD

#### 2.2.1. Parkin: PARK2

Autosomal recessive juvenile parkinsonism (AR-JP) is caused by mutations of the *parkin *gene on chromosome 6q25.2–q27 (PARK2) [12]. The disease is characterized by early onset and a marked response to levodopa treatment and differs from idiopathic PD in that there is usually no LBs formation. Over 100 mutations in *parkin*, including missense mutations and exonic deletions and insertions, have been observed in PD families [41]. Parkin is a ubiquitin E3 ligase preparing target proteins for their degradation mediated by the ubiquitin-proteosomal system [42]. Moreover, parkin is involved in mitochondrial maintenance, repair of mitochondrial DNA damage, might contribute to mitochondrial cytochrome c release, and induce subsequent autophagy of dysfunctional mitochondria [43–47].

#### 2.2.2. *PTEN-Induced Putative Kinase 1 Gene (PINK-1)*: PARK6

Mutations in the *PTEN-induced putative kinase 1 *(*PINK-1*) gene on chromosome 1p35-36 (PARK6) have been linked to autosomal recessive early-onset PD [13]. Several different* PINK1* mutations, primarily missense and nonsense ones, have been identified in PD families worldwide and cause mitochondrial deficits contributing to PD pathogenesis (http://www.molgen.ua.ac.be/PDmutDB/). PINK1 is a kinase with an N-terminal mitochondrial targeting sequence, provides protection against mitochondrial dysfunction, and regulates mitochondrial morphology via fission/fusion machinery. PINK1 also acts upstream of parkin in a common pathway in the maintenance of mitochondrial quality via autophagy [48].

#### 2.2.3. *DJ-1*: PARK7

Mutations in the *DJ-1 *gene on 1p36 (PARK7), including exonic deletions and point mutations, have been associated with a monogenic early-onset autosomal recessive form of parkinsonism characterized by slow progression and response to levodopa [14, 15]. A complete list of DJ-1 mutations can be found at the PD mutation database (http://www.molgen.ua.ac.be/PDmutDB/). DJ-1 is a mitochondrial protein involved in the protection against oxidative stress and forms a complex with parkin and PINK1 to promote ubiquitination and degradation of parkin substrates, including parkin itself [49]. Recent evidence indicates that DJ-1 works in parallel to the PINK1/parkin pathway to maintain mitochondrial function in the presence of an oxidative environment [50].

#### 2.2.4. *ATP13A2* Gene: PARK9

Clinical features similar to those of idiopathic PD and pallydopyramidal syndrome, including parkinsonism, pyramidal tract dysfunction, supranuclear gaze paresis, and dementia, were observed in a Jordanian family. The pattern of transmission was autosomal recessive, and a region of linkage was identified on chromosome 1p36 (PARK9) [16]. The causative gene underlying PARK9 was then identified as the *ATP13A2 *gene encoding a lysosomal 5 P-type ATPase [3] likely involved in the regulation of intracellular manganese homeostasis [51].

### 2.3. Additional PARK Loci

Additional putative PARK loci include: (1) a locus on 2p13, denoted as PARK3 but with no causative gene yet identified; (2) the *UCH-L1* gene on 4p14 (PARK5) coding for a protein that possesses both a hydrolase activity to generate the ubiquitin monomer and a ligase activity to link ubiquitin molecules to tag proteins for disposal; (3) a locus on 1p32, denoted as PARK10 but with no causative gene yet identified; (4) the *GYGYF2 *gene on 2q36-37 (PARK11), encoding a protein that could participate in the regulation of signaling at endosomes; (5) a locus on Xq21-q25, denoted as PARK12 and showing X-linked inheritance, but still pending characterization of the causative gene; (6) the *OMI/HTRA2 *gene on chromosome 2p12 (PARK 13) coding for a nuclear-encoded serine protease localized in the intermembrane space of the mitochondria and involved in mediating caspase-dependent and caspase-independent cellular death; (7) the *PLA2G6 *gene on chromosome 22q13.1 (PARK14) encoding a calcium-independent group VI phospholipase A2; (8) the *FBXO7* gene on 22q11.2-qter (PARK15) encoding for a member of the F-box family of proteins, all of which may have a role in the ubiquitin-proteasome protein-degradation pathway [17–23].

### 2.4. Other Loci

Parkinsonism is often observed as one of the symptoms in other monogenic diseases (Table 2). For example, the *MAPT *gene encodes for the microtubule-associated protein tau, a protein that binds to microtubules and is primarily involved in the organization and integrity of the cytoskeleton. Hyperphosphorylated tau forms neurofibrillary tangles in Alzheimer's disease brains, and mutations of *MAPT *cause frontotemporal dementia with parkinsonism linked to chromosome 17 (FTDP-17) [30, 31]. Parkinsonism, dystonia, and postural tremor are particularly prevalent in spinocerebellar ataxias types 2 (SCA2) and 3 (SCA3). SCA2 can manifest either with a cerebellar syndrome or as Parkinson's syndrome, and SCA3, also known as Machado-Joseph disease (MJD), is the most common form of spinocerebellar ataxia worldwide. The diseases are caused by abnormal CAG trinucleotide repeat expansion of *ataxin-2* (*ATXN2*) and *ataxin-3* (*ATXN3*) genes, respectively [32, 33]. Ataxin-2 is an enzyme involved in RNA processing, whilst ataxin-3 is a deubiquitinating enzyme involved in the ubiquitin-proteasome system. The parkinsonian phenotype of both diseases is often observed in Asians [34, 35]. Autosomal recessive hereditary spastic paraplegia with thin corpus callosum (SPG11) is a rare neurodegenerative disorder often caused by mutations in the gene encoding for spatacsin at the SPG11 locus on chromosome 15q. Two patients, of Turkish descent, from the same consanguineous family, were affected with SPG11 in association with unusual early-onset parkinsonism that occurred during the very early stages of SPG11 in both patients [36]. Additional evidence indicates that mutation of *SPG11* is a rare cause of early-onset levodopa-responsive parkinsonism with pyramidal signs [37]. There is also indication that rearrangements of the gene coding for the mitochondrial DNA polymerase gamma (POLG1), involved in the repair of mitochondrial DNA, can directly cause parkinsonism [38].

## 3. Sporadic Parkinson's Disease

Several hundreds of genetic association studies have been performed in the last few decades by means of the candidate gene approach in order to identify genetic risk factors for non-Mendelian forms of PD.

The candidate gene was selected based on the knowledge of its function in a pathway related to PD pathogenesis, and only one single gene and one or a few polymorphisms were usually investigated. More recently, those studies have been replaced by GWAS where half a million or more polymorphisms are simultaneously investigated in large case-control cohorts. GWASs are currently considered as the gold standard to find loci at which common, normal genetic variability contributes to disease risk, and their introduction has revolutionized our knowledge in the genetics of sporadic PD [24]. The PDGene database [52] is a public and continuously updated database collecting data from PD genetic association studies and GWAS. Accessed on October 2011, the database contained information on 860 studies for a total of 909 candidate genes and 3434 polymorphisms within those genes. In addition, data from GWAS and other large-scale studies were available [52]. There is strong consensus from either GWAS or updated meta-analyses of the literature that variants at four loci (*SNCA, MAPT, GBA* and *LRRK2*) contribute to disease risk (Table 3). In addition, recent GWASs have revealed novel putative PD risk loci to be confirmed in future studies [25], and the meta-analysis of the literature suggests that several additional loci could contribute to disease risk [52] (Table 3).

### 3.1. *α*-Synuclein (*SNCA*)

Common polymorphisms of causative PD genes have been frequently investigated as possible risk factors for the idiopathic forms of the disease. Particularly, genetic polymorphisms in the *SNCA *gene have been consistently associated with PD risk in genetic association studies and subsequently replicated in large-scale GWAS, including a dinucleotide repeat sequence (Rep1) within the promoter region and several single nucleotide polymorphisms (SNPs) at the 3′end of the gene, overall suggesting that *SNCA *alleles associated with increased disease risk correlate with higher *α*-synuclein expression, and pointing to a gene dosage effect [52–60]. Meta-analyses of those studies revealed that *SNCA* is a low-risk locus for idiopathic PD, with odds ratios (ORs) ranging from 1.2 to 1.4 [52].

### 3.2. Leucine-Rich Repeat Kinase 2

Variants of *LRRK2* have been consistently associated with increased risk for sporadic PD in Asians, including a G2385R polymorphism [61] that represents one of the most frequent genetic risk factors for PD in Asian populations, with an estimated OR of 2.2 [52].

### 3.3. Microtubule-Associated Protein Tau: *MAPT*

As described earlier in this paper, mutations of *MAPT* cause frontotemporal dementia with parkinsonism linked to chromosome 17 (FTDP-17). Moreover, large case-control studies, meta-analyses of the literature, and GWAS indicate a role for the* MAPT* haplotype H1 to disease risk [52, 62, 63].

### 3.4. Glucocerebrosidase: *GBA*

Gaucher disease (GD) is an autosomal recessive lysosomal glycolipid storage disorder caused by mutations of the *GBA* gene encoding the enzyme glucocerebrosidase. A small subset of GD patients develop parkinsonism [64] and relatives of patients with GD have an increased incidence of parkinsonism [65]. Large cohort studies [66] and recent meta-analyses of the literature revealed that *GBA* loss of function variants are the most common genetic risk factor associated with parkinsonism, with an estimated OR of 3.4 for the common *GBA* N370S variant [52]. Although the mechanism for this association is unknown, several theories have been proposed, including protein aggregation, lipid accumulation, and impaired autophagy, mitophagy, or trafficking [67].

### 3.5. Additional Loci

The application of GWAS to the understanding of the genetics of sporadic PD has significantly improved our knowledge in the field, and several loci have been associated with disease risk in recent years. A recent meta-analysis of published GWAS was performed by the International Parkinson Disease Genomics Consortium (IPDGC) for a total of 12,386 PD cases and 21,026 controls and suggested that, in addition to* SNCA*, *LRRK2, *and *MAPT *polymorphisms, variants at eight additional loci (*HLA-DRB5, BST1, GAK, ACMSD, STK39, MCCC1/LAMP3, SYT11, *and* CCDC62/HIP1R*) are significantly associated with disease risk [25]. A more recent two-stage meta-analysis performed by the IPDGC and the Wellcome Trust Case Control Consortium 2 (WTCCC2) revealed five additional loci associated with PD risk (*PARK16/1q32*, *STX1B*, *GWA 8p22*, *STBD1*, *GPNMB*) [68]. Up to date meta-analyses are available also at the PDGene database for each polymorphism that has been evaluated in at least four independent genetic association studies. Accessed on October 2011 the PDGene database contained 886 updated meta-analyses of the literature, overall suggesting that 18 loci could contribute to sporadic PD risk, including most of those already described (*SNCA, LRRK2, MAPT, GBA, HLA-DRB5, BST1, GAK, ACMSD, STK39, MCCC1/LAMP3, SYT11, CCDC62/HIP1R*, *GWA 8p22*, *GPNMB*) and additional ones (*PM20D1, SETD1A, FAM47E *and *MED13*) [52].

## 4. Epigenetics of Parkinson's Disease

### 4.1. DNA Methylation

DNA methylation represents one of the most important epigenetic processes, along with histone modifications and mechanisms involving small RNA molecules. Methylation of CpG sequences might induce chromatin conformational modifications and inhibit the access of the transcriptional machinery to gene promoter regions, thus altering gene expression levels. Therefore, promoter hypermethylation is commonly associated with gene silencing and promoter demethylation with gene expression, even if some exceptions to this rule are known [69]. Folate, other B vitamins (vitamins B6 and B12), and homocysteine (hcy) participate in one-carbon metabolism, a complex pathway required for the production of S-adenosylmethionine (SAM), the major intracellular methylating agent. DNA methylation is closely dependent on the DNA methylation potential, which is referred to as the ratio between SAM and S-adenosylhomocysteine (SAH) levels. DNA methyltransferases (DNMTs) are the key enzymes for DNA methylation and catalyze the transfer of a methyl group from SAM to cytosine, thus forming 5-methyl-cytosine. Impaired one-carbon metabolism, altered DNA methylation potential, reduced DNA methylation levels in the brain, and altered methylation and expression of several genes were observed in Alzheimer's disease (AD) patients, pointing to a role for epigenetic modifications in the neurodegenerative process [26]. Less studies have been so far performed in PD subjects; however, there is indication of impaired one-carbon metabolism in PD as well as altered DNA methylation potential [70, 71]. Epigenetic analyses of PD brains revealed that the *SNCA *gene could be subjected to epigenetic regulation [72, 73]. In addition, more recent large-scale studies suggest that other PD-related genes could be epigenetically modified in PD brains (Table 4) [68].

#### 4.1.1. *α*-Synuclein

Several lines of evidence, including the identification of families with *SNCA* locus duplication and triplication and the association of both promoter and 3′UTR polymorphisms with sporadic forms, point to a gene-dosage effect for *SNCA *in PD pathogenesis [9]. Studies in individuals with alcoholism [74] and in anorexia patients [75] revealed hypermethylation of the *SNCA* promoter, suggesting that the gene could be epigenetically regulated. The analysis of *SNCA* alleles in a PD patient heterozygous for the A53T mutation, the first mutation to be implicated in PD pathogenesis, revealed that *SNCA* showed monoallelic expression in this patient, with epigenetic silencing of the mutated allele due to histone modifications but not DNA methylation, and upregulation of the wild-type allele resulting in higher mRNA levels than in matched control subjects [76]. Others observed that the methylation of human *SNCA* intron 1 decreases gene expression, while inhibition of DNA methylation activates *SNCA* expression. They also observed that DNA methylation of *SNCA* intron 1 was reduced in several brain regions of sporadic PD patients, including the *substantia nigra*, putamen, and cortex, pointing toward an epigenetic regulation of *SNCA *expression in PD [72]. Another research group identified an *SNCA* CpG island in which the methylation status altered along with increased *SNCA* expression. Postmortem brain analysis revealed regional nonspecific methylation differences in this CpG region in the anterior cingulate and putamen among controls and PD subjects; however, in the *substantia nigra* of PD individuals, the methylation of this region was significantly decreased [73]. Both findings are consistent with previous reports indicating increased *SNCA *mRNA levels in PD *substantia nigra* tissue [77, 78]. A recent paper suggested that *α*-synuclein sequesters DNA methyltransferase 1 (DNMT1) from the nucleus [79]. DNMT1 is the maintenance DNA methylation enzyme which is abundantly expressed in the adult brain and is mainly located in the nuclear compartment. The researchers observed a reduction of nuclear DNMT1 levels in human postmortem brain samples from PD and from patients with dementia with Lewy bodies (DLBs) as well as in the brains of *α*-synuclein transgenic mice models. Furthermore, sequestration of DNMT1 in the cytoplasm resulted in global DNA hypomethylation in human and mouse brains, involving CpG islands upstream of *SNCA* and other genes. The nuclear DNMT1 levels were partially rescued by overexpression of DNMT1 in neuronal cell cultures and in *α*-synuclein transgenic mice brains. Therefore, the authors suggested that the association of DNMT1 and *α*-synuclein might mediate aberrant subcellular localization of DNMT1, resulting in epigenetic modifications in the brain [79].

#### 4.1.2. Other Genes

In addition to its association with ARJP, loss of heterozygosity of *parkin* has been found in several types of malignant tumors, including ovarian, breast, and hepatocellular tumors, and abnormal methylation of *parkin* promoter was observed in patients with cancer [80]. The levels of methylation of the *parkin* gene promoter were revealed in samples from PD patients with heterozygous parkin mutations, PD patients without parkin mutations, and normal controls. No difference was observed between the three groups, suggesting that *parkin *promoter methylation is unlikely to play a role in the pathogenesis and development of PD [101]. The *UCHL-1 *gene promoter was found to be hypermethylated in various types of cancer [102, 103]. However, the analysis of *UCHL-1 *promoter methylation in the frontal cortex of PD patients and controls revealed no significant differences between the groups [104]. In the same study the authors evaluated the methylation profiles of *MAPT *promoter in the frontal cortex and hippocampus of controls, Alzheimer's disease patients, PD patients, and subjects with other tauopathies and synucleinopathies. No differences in the percentage of CpG methylation were found between control and disease samples or among the different pathological entities in any region analyzed [104]. Another study reported methylation differences in the gene coding for tumor necrosis alpha (*TNFA*) between the cortex and the *substantia nigra*, but these differences were present in PD cases and controls [105]. The analysis of the original Chilean family with Kufor-Rakeb syndrome that led to the discovery of the *ATP13A2* gene at the PARK9 locus also revealed that there was no significant correlation between DNA methylation of the *ATP13A2 *promoter region and disease progression [106]. However, the recent collaborative study of the IPDGC and WTCCC2 on a dataset of post-mortem brain samples assayed for gene expression (*n* = 399) and methylation (*n* = 292), revealed methylation and expression changes associated with PD risk variants in *PARK16/1q32*, *GPNMB*, and *STX1B*, suggesting that the *SNCA* gene is unlikely to be the only one subjected to epigenetic regulation in PD brains [68]. In addition, an aging-associated alteration of subtelomeric methylation patterns was observed in peripheral leukocytes of Japanese PD patients that showed fewer short telomeres than healthy controls. Moreover, short telomeres in PD patients showed a constant methylation pattern, whilst an age-related demethylation of short telomeres was observed in controls [100].

### 4.2. Histone Modifications

Gene expression profiles are modulated not only by promoter methylation but also by the chromatin state. Indeed, chromatin can exist in a condensate inactive state (heterochromatin) or in a noncondensate and transcriptionally active state (euchromatin). Conformational changes in histone proteins or modifications of the way in which DNA wraps around the histone octamer in nucleosomes may either alter or facilitate the access of the transcriptional machinery to the promoter region of some genes, leading to gene silencing or activation, respectively. Histone tail modifications include acetylation, methylation, phosphorylation, ubiquitylation, sumoylation, and other posttranslational modifications. Histone tail acetylation is associated with chromatin relaxation and transcriptional activation, while deacetylation is related to a more condensed chromatin state and transcriptional repression [107]. Histone acetyltransferases (HATs) catalyze the acetylation of lysine residues in histone tails, whereas histone deacetylation is mediated by histone deacetylases (HDACs). Another frequently studied modification of histone tails is methylation on either lysine or arginine residues mediated by histone methyltransferases. Methylation of histone tails can be associated with either condensation or relaxation of the chromatin structure, since several sites for methylation are present on each tail thus allowing several combinations [107, 108]. Little is still known concerning histone modifications in PD brains and most of the current knowledge is derived from studies in cell cultures and animal models of the disease, such as those induced by mitochondrial toxins, including 1-methyl-4-phenylpyridinium (MPP+), paraquat, and rotenone, or those overexpressing human *α*-synuclein (Table 4). However, those studies have revealed that *α*-synuclein interacts with histones and inhibits histone acetylation [81, 82] and that several histone deacetylase inhibitors (HDACIs) are neuroprotective against *α*-synuclein-mediated toxicity [82, 84–87]. Particularly, studies performed in nigral neurons of mice exposed to the herbicide paraquat revealed that *α*-synuclein translocates into the nucleus and binds with histones [81]. Studies in *Drosophila *showed that *α*-synuclein mediates neurotoxicity in the nucleus, binds directly to histone H3, and inhibits histone acetylation. The toxicity of *α*-synuclein was rescued by the administration of HDACIs [82]. The inhibition of the histone deacetylase Sirtuin 2 rescued *α*-synuclein-mediated toxicity in several models of PD [84]. In addition valproic acid (VPA) resulted in inhibition of histone deacetylase activity and in an increase of histone H3 acetylation in brain tissues of rats and resulted neuroprotective in a rat model of PD (obtained with the administration of the mitochondrial toxin rotenone), counteracting *α*-synuclein translocation into the nuclei [85]. Studies in rat and human neuronal cell cultures also revealed that HDACIs prevent MPP+ -mediated cytotoxicity [86]. Neurotoxic pesticides and paraquat were shown to increase histone acetylation in mice brains or cell culture models [87, 88], and additional studies reported protective effects of HDACIs on dopaminergic neurons following a neurotoxic-induced insult [89, 90]. A recent study in *α*-synuclein transgenic mice revealed that *α*-synuclein negatively regulates protein kinase C*δ* expression to suppress apoptosis in dopaminergic neurons by reducing p300 histone acetyltransferase activity [83]. A genome-wide expression screen was performed in *C. elegans *overexpressing human *α*-synuclein, and nine genes that form histones H1, H2B, and H4 were downregulated [91]. Overall, these studies point to a role for histone modifications in *α*-synuclein-mediated as well as environmental-induced dopaminergic neuronal cell death. Moreover, the accumulation of misfolded proteins in proteinaceous inclusions termed aggresomes is a cytoprotective response serving to sequester potentially toxic misfolded proteins and facilitate their clearance by autophagy. Histone deacetylase 6 (HDAC6) plays a fundamental role in aggresome formation. HDAC6 is concentrated in Lewy bodies in PD and dementia with LBs, and the *Drosophila *histone deacetylase 6 (dHDAC6) was shown to play a critical role in the protection of dopaminergic neurons and promoted the formation of *α*-synuclein inclusions in a *Drosophila *PD model expressing human *α*-synuclein. On the contrary, mutation of dHDAC6 resulted in the accumulation of toxic *α*-synuclein oligomers [109]. It was suggested that the accumulation of HDAC6 might be specific to *α*-synucleinopathy and that LBs might represent cytoprotective responses to sequester toxic proteins [110]. Aggresome formation involves several regulators, including HDAC6, parkin, ataxin-3, and ubiquilin-1 [111]. It has been recently shown in neuronal cell cultures that HDAC6 participates in the degradation of MPP+ induced aggregates of *α*-synuclein by regulating the aggresome-autophagy pathway [112].

### 4.3. RNA-Mediated Epigenetic Mechanisms

MicroRNAs (miRNAs) are a group of small noncoding RNAs that bind to the 3′ untranslated region (3′UTR) of target mRNAs and mediate their posttranscriptional regulation leading to either degradation or translational inhibition, depending on the degree of sequence complementarity. In 2007 Kim et al. investigated the role of miRNAs in mammalian midbrain dopaminergic neurons and identified an miRNA, miR-133b, that is specifically expressed in midbrain dopaminergic neurons and is deficient in midbrain tissue from patients with PD. miR-133b regulates the maturation and function of midbrain dopaminergic neurons within a negative feedback circuit that includes the paired-like homeodomain transcription factor Pitx3 [97]. Subsequently, others observed that the *FGF20 *rs12720208 polymorphism disrupts a binding site for miR-433, increasing translation of *FGF20 in vitro* and *in vivo*, and suggested that this could represent a risk factor for PD [113]. However, a recent study failed to confirm the association between rs12720208 and PD risk, or any effect of miR-433 variants to PD pathogenesis [114]. MicroRNA expression analysis in brains of early symptomatic *α*-synuclein(A30P)-transgenic mice showed that the levels of several miRNAs (miR-10a, -10b, -212, -132, -495) were significantly altered. MiR-132 was reported to be highly inducible by growth factors and to be a key regulator of neurite outgrowth [92]. Others observed that miR-7, which is expressed mainly in neurons, represses *α*-synuclein protein levels binding to *α*-synuclein mRNA. Further, miR-7 expression decreased in MPP+ -induced models of PD in cultured cells and in mice, thereby contributing to increased *α*-synuclein expression [93]. Another study confirmed that mir-7 regulates *α*-synuclein levels together with mir-153. Both miRNAs bind specifically to the 3′-untranslated region of *α*-synuclein and downregulate its mRNA and protein levels, with their effect being additive. They are expressed predominantly in the brain with a pattern that mirrors synuclein expression in different tissues as well as during neuronal development, likely playing a tuning role in the amount of *α*-synuclein produced [94]. The analysis of *C. elegans *models of PD revealed that several miRNAs were underexpressed in those animals; particularly the family of miR-64 and miR-65 were co-underexpressed in *α*-synuclein transgenic animals, and members of let-7 family co-underexpressed in both *α*-synuclein and parkin strains [95]. A recent study demonstrates that blood samples can be used as a source of miRNAs associated to PD. Six differentially expressed miRNAs were identified. While miR-1, miR-22*, and miR-29 expression levels allowed to distinguish nontreated PD patients from healthy subjects, miR-16-2*, miR-26a2*, and miR30a differentiated treated from untreated PD patients [99]. A recent miRNA profiling of PD brains identified early downregulation of miR-34b/c which modulate mitochondrial function. Particularly, misregulation of miR-34b/c was detected in premotor stages (stages 1–3) of the disease, and thus in cases that did not receive any PD-related treatment during life, and miR-34b/c downregulation was coupled to a decrease in the expression of DJ1 and Parkin proteins [98]. Moreover, studies in *Drosophila *bearing LRRK2 PD-associated mutations revealed that pathogenic LRRK2 antagonizes let-7 and miR-184*, leading to the overproduction of the E2F1/DP complex involved in cell cycle and survival control [96]. Overall, these studies indicate microRNA-mediated mechanisms in PD pathogenesis (Table 4).

## 5. Conclusions

Several advances have been gained in our understanding of the genetics of PD since the first report [5] of a *SNCA *gene mutation causing familial autosomal dominant PD in 1997 (Table 1). Studies on familial recessive forms of the disease have highlighted a central role for mitochondrial damage, repair, and turnover in the pathophysiology of the disease, with *parkin*, *DJ1*, *PINK1*, and *FBX07*, participating in the same or in similar/overlapping mitochondrial pathways. In addition, as suggested by Hardy [24], both glucocerebrosidase and ATP13A2 are lysosomal enzymes, indicating a second PD pathway involving lysosomes. In contrast, *α*-synuclein and LRRK2 biology is still poorly understood, despite that both proteins are central to the disease etiology [24]. Association studies in sporadic cases and the recent application of genome-wide technology led to the identification of almost 20 genes that could modify the individual risk for the idiopathic forms of the disease (Table 3), and additional genes are expected to be unravelled within the next future. Public databases, such as the PD mutation database (http://www.molgen.ua.ac.be/PDmutDB/) and the PDGene database [52], have been created to continuously update the genetics of either familial or idiopathic forms, respectively. There is however a growing evidence that, in addition to genetic mutations, also epigenetic mechanisms could contribute to disease pathogenesis (Table 4). For example, studies in PD families as well as in sporadic PD cases suggest an *SNCA *gene-dosage effect critical to disease pathogenesis, but there is also indication of upregulated *SNCA *gene expression resulting from promoter demethylation in PD brains [72, 73]. *α*-Synuclein itself was shown to exert epigenetic properties, such as histone tail modifications [81, 82, 84, 85] and DNMT1 sequestration [79]. In addition, microRNAs were found to modulate *α*-synuclein expression [93, 94]. Other genes critical to PD pathogenesis have been found to be regulated by promoter methylation [68] or RNA-mediated mechanisms [98]. For example, a decrease in the expression of DJ1 and parkin proteins can result from microRNA-mediated mechanisms in PD brains, ultimately leading to mitochondrial impairments such as those caused by *parkin *or *DJ-1 *gene mutations [98]. The increasing amount of papers aimed at understanding the epigenetics of PD has led to a better understanding of the molecular pathways involved in dopaminergic neuron degeneration, and several researchers are now working to understand the therapeutic potentials of epigenetic molecules to counteract age-related neurodegenerative diseases [115, 116]. Future research in PD should be aimed at understanding the complex interplay among genetic and epigenetic biomarkers, lifestyles, and environmental factors, to further characterize individuals at risk to develop the disease.

## Figures and Tables

**Table 1:** Loci and genes associated with familial PD.

Designation	Locus	Gene	Inheritance*	Refs
*Validated loci*				
PARK1/PARK4	4q21	*SNCA*	AD	[5–16]
PARK8	12q12	*LRRK2*	AD	
PARK2	6q25.2–q27	*PARK*	AR	
PARK6	1p35-36	*PINK1*	AR	
PARK7	1p36	*DJ-1*	AR	
PARK9	1p36	*ATP13A2*	AR	
*Other loci*				
PARK3	2p13	Unknown	AD	[17–23]
PARK5	4p14	*UCH-L1*	AD	
PARK8	12q12	*LRRK2*	AD	
PARK10	1p32	Unknown	Not clear	
PARK11	2q36-37	*GIGYF2*	AD	
PARK12	Xq21-q25	Unknown	X-linked	
PARK13	2p12	*OMI/* *HTRA2*	AD	
PARK 14	22q13.1	*PLA2G6*	AR	
PARK 15	22q11.2-qter	*FBXO7*	AR	

*AD: autosomal dominant; AR: autosomal recessive.

**Table 2:** Additional genes causing parkinsonism.

Gene	Disease	Refs
*MAPT *	Frontotemporal dementia with parkinsonism linked to chromosome 17	[30–38]
*ATXN2*	Spinocerebellar ataxia type 2 and parkinsonism	
*ATXN3*	Spinocerebellar ataxia type 3 and parkinsonism	
*SPG11*	Hereditary spastic paraplegia and parkinsonism	
*POLG1*	Mitochondrial parkinsonism	

**Table 3:** Genes or loci associated with idiopathic PD.

Gene or locus	Methodologies employed	Refs
*SNCA *	Large-scale association studies	[25, 52–68]
*LRRK2 *		
*MAPT *	Meta-analyses of genetic association studies	
*GBA *		
*HLA-DRB5 *		
*BST1 *	GWAS	
*GAK *		
*ACMSD *	Meta-analyses of GWAS	
*STK39 *		
*MCCC1/LAMP3*		
*SYT11 *		
*CCDC62/HIP1R *		
*PARK16/1q32 *		
*STX1B*		
*GWA 8p22*		
*STBD1*		
*GPNMB *		
*PM20D1 *		
*SETD1A *		
*FAM47E *		
*MED13*		

**Table 4:** Epigenetic changes of PD related genes or PD tissues.

*Gene*	Observation	Refs
	Reduced *SNCA *methylation in the *substantia nigra *of PD patients	[72, 73]
	*α*-synuclein sequesters DNMT1 from the nucleus resulting in aberrant DNA methylation	[79]
	*SNCA *gene silencing mediated by histone modifications	[76]
	*α*-synuclein binds to histones and inhibits histone acetylation	[81–83]
**α*-synuclein (SNCA)*	Histone deacetylase inhibitors are neuroprotective against *α*-synuclein mediated neurotoxicity in PD animal models	[82, 84–90]
	*α*-synuclein expression in *C. elegans *results in down-regulation of genes coding for histones	[91]
	miR-10a, -10b, -212, -132, -495 were impaired in presymptomatic **α*-synuclein *transgenic mice	[92]
	miR-7 and mir-153 regulates *α*-synuclein levels	[93, 94]
	miR-64 and mir-65 and let-7 were co-under-expressed in **α*-synuclein *transgenic *C. elegans *	[95]

*LRRK2*	Mutant LRRK2 antagonizes miR-184* and let7 in *Drosophila *PD models	[96]

*parkin*	let-7 family miRNAs let-7 were co-under-expressed in *parkin *transgenic *C. elegans *	[95]

*PARK16/1q32, GPNMB, STX1B*	Aberrant gene methylation observed in post-mortem PD brains	[68]

*Tissue*	Observation	Refs

PD brains	miR-133b was deficient in midbrain from PD patients	[97]

PD brains	miR-34b/c down-regulation was observed in pre-motor stages of PD and resulted in altered expression of DJ1 and parkin proteins	[98]

PD lymphocytes	Altered expression of miR-1, miR-16-2*, miR-22*, miR26a2*, miR29, miR30	[99]

PD leukocytes	Altered methylation patterns of subtelomeric regions	[100]
